# MRI super-resolution reconstruction using efficient diffusion probabilistic model with residual shifting

**Published:** 2025-03-03

**Authors:** Mojtaba Safari, Shansong Wang, Zach Eidex, Qiang Li, Erik H. Middlebrooks, David S. Yu, Xiaofeng Yang

**Affiliations:** 1 Department of Radiation Oncology and Winship Cancer Institute, Emory University, Atlanta, GA 30322, United States.; 2 Department of Radiology, Mayo Clinic, Jacksonville, FL, United States.

**Keywords:** Super-resolution, MRI, Deep learning, Reconstruction, Diffusion model, Brain T1 map, Ultra-high field MRI

## Abstract

**Objective:**

MRI offers superior soft-tissue contrast yet suffers from long acquisition times that can induce patient discomfort and motion artifacts. Super-resolution (SR) methods reconstruct high-resolution (HR) images from low-resolution (LR) scans, but diffusion models typically require numerous sampling steps, hindering real-time use. Here, we introduce a residual error-shifting strategy that reduce sampling steps without compromising anatomical fidelity, thereby improving MRI SR for clinical deployment.

**Approach:**

We propose a novel diffusion-based SR framework called Res-SRDiff, which integrates residual error shifting into the forward diffusion process. This approach enables efficient HR image reconstruction by aligning the degraded HR image distribution with the LR image distribution. Our model was evaluated on two MRI datasets: ultra-high-field brain T1 MP2RAGE maps and T2-weighted prostate images. We compared Res-SRDiff against established methods, including Bicubic, Pix2pix, CycleGAN, and a conventional denoising diffusion probabilistic model with vision transformer backbone (TM-DDPM), using quantitative metrics such as peak signal-to-noise ratio (PSNR), structural similarity index (SSIM), gradient magnitude similarity deviation (GMSD), and learned perceptual image patch similarity (LPIPS).

**Main results:**

Res-SRDiff significantly outperformed all comparative methods in terms of PSNR, SSIM, and GMSD across both datasets, with statistically significant improvements (p-values ≪ 0.05). The model achieved high-fidelity image restoration with only four sampling steps, drastically reducing computational time to under one second per slice, which is substantially faster than conventional TM-DDPM with around 20 seconds per slice. Qualitative analyses further demonstrated that Res-SRDiff effectively preserved fine anatomical details and lesion morphology in both brain and pelvic MRI images.

**Significance:**

Our findings show that Res-SRDiff is an efficient and accurate MRI SR method, markedly improving computational efficiency and image quality. By integrating residual error shifting into the diffusion process, it allows for rapid and robust HR image reconstruction, enhancing clinical MRI workflows and advancing medical imaging research. The source at: https://github.com/mosaf/Res-SRDiff

## 1 Introduction

Magnetic resonance imaging (MRI) is an indispensable tool in both clinical practice and research, providing detailed anatomical and functional images. Quantitative techniques, such as 3D magnetization-prepared 2 rapid acquisition gradient echo (MP2RAGE) T1-maps, offer robust imaging free from reception bias and first-order transmit field inhomogeneities, thereby enabling precise diagnosis and treatment planning [1–3]. For example, T1-maps are employed to identify hypoxic regions that can inform adaptive dose-painting radiation therapy [4–6]. Moreover, in addition to these quantitative methods, T2-weighted (T2w) MRI provides enhanced tissue contrast, rendering it a critical imaging modality for prostate cancer treatment by delineating tumor boundaries and guiding therapeutic decisions [7]. Nevertheless, the lengthy acquisition times associated with both T1-mapping and T2w imaging may induce patient discomfort and elevate the risk of motion artifacts [8], thereby potentially compromising image quality and diagnostic accuracy.

To accelerate MRI image acquisition, super-resolution (SR) studies have aimed to reconstruct high-resolution (HR) images from their low-resolution (LR) counterparts [9]. Conventional SR models, which constitute a subcategory of the broader field of image restoration, employ a maximum a posteriori framework–a Bayesian paradigm consisting of a likelihood (loss) function and a prior (regularization) term–to resolve the ill-posed SR task. The likelihood term presupposes an underlying noise distribution, yielding ${𝓛}_{2}$ and ${𝓛}_{1}$ losses for Gaussian and Laplacian noise assumptions, respectively. Typical regularizers include Tikhonov [10], non-local similarity [11], wavelet [12], and total variation [13] to address the ill-posed image restoration task.

However, deep learning algorithms, especially generative deep learning models, exhibit superior performance to conventional algorithms in medical imaging tasks such as reconstruction [14, 15] and denoising [16]. Among them, generative diffusion models have been successfully used in MRI image reconstruction [17], denoising [18], and super-resolution [19–21]. Diffusion models consist of forward and backward processes, where the former is constructed using a Markov chain to convert data to a predetermined prior distribution, such as the multivariate standard Gaussian distribution 𝒩(0,I), and the latter trains a neural network (NN) to approximate the inverse trajectory using the Markov chain. In the sampling step, the trained NN generates images by randomly sampling from the reverse Markov chain, starting from 𝒩(0,I) over numerous sampling steps T. These diffusion models have two drawbacks for the image SR task. First, due to their iterative sampling process, they are inefficient for generating HR images from LR image pairs. Second, initiating reconstruction from 𝒩(0,I) is more appropriate for image generation than for restoration tasks. Yue *et al.* [22] argued the inefficiency of this process for image restoration tasks, including SR and denoising. It has been demonstrated that initializing the image reconstruction with a distribution centered around the LR image, rather than Gaussian noise, facilitates a more efficient sampling process [23], achieved by shifting the residual errors between LR and HR images over T steps.

In this study, we present an efficient diffusion model that exploits the residual error shift between HR and LR image pairs to reconstruct HR axial T2w prostate images and quantitative brain MRI T1 MP2RAGE maps obtained from ultra-high ${\text{B}}_{0}$ fields, extending the work presented in [22, 23]. We refer to this efficient diffusion model as “Res-SRDiff” throughout this paper. To our knowledge, this is the first investigation aimed at recovering HR MRI using an efficient diffusion model that requires **only four sampling steps**, in contrast to the thousands required by conventional diffusion models. This substantial reduction in sampling steps markedly enhances the model’s efficiency without compromising the quality of the restored HR images.

The contributions of this work are:

- We formulate an efficient diffusion model for the SR task, enabling inference in only four sampling steps.
- We employed a U-net model that utilized a Swin Transformer block instead of an attention layer to better generalize across different image resolutions.
- We extensively evaluated our model using public axial T2w prostate images and institutional ultra-high 7T T1 MP2RAGE maps.
- To the best of our knowledge, this study is the first to employ an efficient diffusion model to reconstruct HR axial T2w pelvic images and ultra-high ${\text{B}}_{0}$ field brain T1 maps from LR pairs using diffusion techniques.

## 2 Materials and Methods

In this section, we first review the traditional Denoising Diffusion Probabilistic Model (DDPM). Next, we introduce our proposed method, Res-SRDiff, which is designed to recover HR images $\left(x^{\text{HR}}\right)$ from their LR counterparts $\left(x^{\text{HR}}\right)$. We assume that both HR and LR images have similar spatial size, an assumption that can be readily satisfied by pre-upsampling the LR images using nearest neighbor interpolation.

### 2.1 DDPM

The DDPM was initially inspired by non-equilibrium thermodynamics [24], aiming to approximate a complex data distribution with a tractable distribution, such as a standard Gaussian distribution. It was later enhanced by integrating stochastic differential equations and denoising score matching [25, 26]. The DDPM comprises two diffusion processes: a forward process and a reverse process. The forward process degrades the input image into noise following a standard Gaussian distribution 𝒩(0,I) over numerous steps T. The reverse process trains an NN to approximate the sampling trajectory required to recover the input image from Gaussian noise over a large number of steps T, which diminishes the sampling efficiency of the DDPM.

### 2.2 Problem formulation

**Figure 1:**
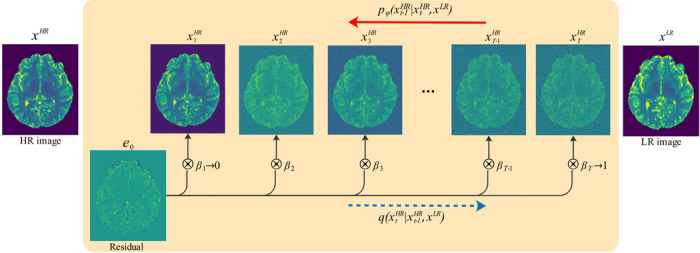
Illustration of the forward diffusion process in Res-SRDiff, where a HR image $x^{\text{HR}}$ is progressively shifted to match the LR distribution $q\left(x^{\text{LR}}\right)$. The model introduces a residual error $e_{0}=x^{\text{LR}}-x^{\text{HR}}$, which drives $x^{\text{HR}}$ through T Markov steps until $q\left(x_{T}^{\text{HR}}\right)\approx q\left(x^{\text{LR}}\right)$, rather than converging to a standard Gaussian distribution.

Res-SRDiff is built upon a Markov chain, similar to the conventional DDPM model. However, it aims to degrade input HR images $x^{\text{HR}}$ into an image $x_{T}^{\text{HR}}$ over T steps such that the resulting distribution $q\left(x_{T}^{\text{HR}}\right)$ approximates $q\left(x^{\text{LR}}\right)$ rather than converging to 𝒩(0,I). This is achieved by introducing the residual $e_{0}=x^{\text{LR}}-x^{\text{HR}}$, which is used to shift $x^{\text{HR}}$ over the T steps. This process is illustrated in Figure 1.

#### Forward process.

To simulate the forward diffusion process, a monotonically increasing shifting sequence ${\beta_{t}}_{t=1}^{T}$ over time steps t with bounding conditions $\beta_{1}\to 0$ and $\beta_{T}\to 1$ is used. The transition kernel for simulating the forward diffusion process is given in (1), which is constructed based on the Markov chain and the residual error $e_{0}$ shift sequences (see Figure 1):

(1) $$q\left(x_{t}^{\text{HR}}|x_{t-1}^{\text{HR}},x^{\text{LR}}\right)=𝒩\left(x_{t}^{\text{HR}};x_{t-1}^{\text{HR}}+e_{0}\alpha_{t},\gamma^{2}\alpha_{t}\text{I}\right),\;t\in [1,T]$$

where $\alpha_{1}=\beta_{1}\to 0$ and $\alpha_{t}=\beta_{t}-\beta_{t-1}$ for t>1, and γ is a hyper-parameter introduced to improve the flexibility of the forward diffusion process. Considering the Markov chain, we can compute the image at step t from its step t−1 using the reparameterization trick as follows:

(2) $$x_{t}=x_{t-1}+\alpha_{t}e_{0}+\sqrt{\gamma^{2}\alpha_{t}\epsilon }$$

Since this sampling forward process using (2) increases the computational burden, it is also possible to compute the image at step t directly from the input noise-free image as follows:

(3a) $$x_{1}=x_{0}+\alpha_{1}e_{0}+\sqrt{\gamma^{2}\alpha_{1}\epsilon }$$

(3b) $$x_{2}=x_{1}+\alpha_{2}e_{0}+\alpha_{1}\epsilon_{0}+\sqrt{\gamma^{2}\alpha_{2}}\epsilon$$

(3c) $$=x_{0}+\left(\alpha_{1}+\alpha_{2}\right)e_{0}+\gamma \left(\sqrt{\alpha_{1}}+\sqrt{\alpha_{2}}\right)\epsilon \;\vdots$$

(3d) $$x_{t}=x_{0}+e_{0}\sum_{t^{\prime }=1}^{T}\alpha_{t^{\prime }}+\gamma \left(\sum_{t^{\prime }=1}^{T}\sqrt{\alpha_{t^{\prime }}}\right)\epsilon$$

Here, we omit the superscript HR for brevity. The second term (mean) and the square of the third term (variance) in the summation given in (3d) are equal to $\beta_{t}$. Thus, the marginal distribution at any time step t can be computed analytically as follows:

(4) $$p\left(x_{t}^{\text{HR}}|x^{\text{HR}},x^{\text{LR}}\right)=𝒩\left(x_{t}^{\text{HR}};x^{\text{HR}}+e_{0}\beta_{t},\gamma^{2}\beta_{t}\text{I}\right),\;t\in [1,T]$$

#### Reverse process.

The reverse process trains a NN $g_{\varphi }$ to estimate the posterior distribution $p\left(x^{\text{HR}}|x^{\text{LR}}\right)$, as follows [27]:

(5) $$p\left(x^{\text{HR}}|\text{LR}\right)=\int p\left(x_{T}^{\text{HR}}|x^{\text{LR}}\right)\prod_{t=1}^{T}p_{\varphi }\left(x_{t-1}^{\text{HR}}|x_{t}^{\text{HR}},x^{\text{LR}}\right)dx_{1:T}$$

where $p\left(x_{T}^{\text{HR}}|x^{\text{LR}}\right)\approx 𝒩\left(x^{\text{HR}};x^{\text{LR}},\gamma^{2}\text{I}\right)$ and $p_{\varphi }\left(x_{t-1}^{\text{HR}}|x_{t}^{\text{HR}},x^{\text{LR}}\right)$) is a reverse transition kernel that aims to learn $x_{t-1}^{\text{HR}}$ from $x_{t}^{\text{HR}}$ by training a network $g_{\varphi }$. Similar to conventional diffusion models [25–27], it can be written as follows by adopting the Gaussian assumption:

(6) $$p_{\varphi }\left(x_{t-1}^{\text{HR}}|x_{t}^{\text{HR}},x^{\text{LR}}\right)=𝒩\left(x_{t-1}^{\text{HR}};\mu_{\varphi }\left(x_{t}^{\text{HR}},x^{\text{LR}},t\right),{\text{Σ}}_{\varphi }(t)\right)$$

where the optimum parameter φ is achieved by minimizing the Kullback-Leibler (KL) divergence between the forward and reverse kernels summed over all time steps as follows [27]:

(7) $$\underset{\varphi }{\arg\min}\sum_{t}D_{KL}\left(q\left(x_{t-1}^{\text{HR}}|x_{t}^{\text{HR}},x^{\text{HR}},x^{\text{LR}}\right)|p_{\varphi }\left(x_{t-1}^{\text{HR}}|x_{t}^{\text{HR}},x^{\text{LR}}\right)\right)$$

The target distribution $q\left(x_{t-1}^{\text{HR}}|x_{t}^{\text{HR}},x^{\text{HR}},x^{\text{LR}}\right)$ can be computed using (1) and (4), along with the Markov chain assumption, which states $x_{t}⊥x_{1:t-2}|x_{t-1}$, as follows:

(8) $$q\left(x_{t-1}^{\text{HR}}|x_{t}^{\text{HR}},x^{\text{HR}},x^{\text{LR}}\right)=q\left(x_{t}^{\text{HR}}|x_{t-1}^{\text{HR}},x^{\text{LR}}\right)q\left(x_{t-1}^{\text{HR}}|x^{\text{HR}},x^{\text{LR}}\right)\;\;\;\;\;\;\;\;\;\;\;=𝒩\left(x_{t}^{\text{HR}};x_{t-1}^{\text{HR}}+\alpha_{t}e_{0},\gamma^{2}\alpha_{t}\text{I}\right)𝒩\left(x_{t-1}^{\text{HR}};x^{\text{HR}}+e_{0}\beta_{t-1},\gamma^{2}\beta_{t-1}\text{I}\right)$$

The multiplication of two Gaussian distributions yields another Gaussian distribution that can be computed tractably [28] as follows:

(9) $$q\left(x_{t-1}^{\text{HR}}|x_{t}^{\text{HR}},x^{\text{HR}},x^{\text{LR}}\right)=𝒩(x_{t-1}^{\text{HR}};\underset{\mu_{q}}{\underset{︸}{\frac{\beta_{t-1}}{\beta_{t}}x_{t}^{\text{HR}}+\frac{\alpha_{t}}{\beta_{t}}x^{\text{HR}}}},\underset{{\text{Σ}}_{q}}{\underset{︸}{\gamma^{2}\alpha_{t}\frac{\beta_{t-1}}{\beta_{t}}\text{I}}})$$

By assuming that the forward and backward covariance matrices are similar $({\text{Σ}}_{q}={\text{Σ}}_{\varphi })$, the KL divergence given in (7) simplifies to:

(10) $$\hat{\varphi }=\underset{\varphi }{\arg\min}\frac{1}{2}\left[{\left(\mu_{\varphi }-\mu_{q}\right)}^{T}{\text{Σ}}_{q}{\left(t\right)}^{-1}\left(\mu_{\varphi }-\mu_{q}\right)\right]\;\;=\underset{\varphi }{\arg\min}\frac{1}{2}\left[{\left(\mu_{\varphi }-\mu_{q}\right)}^{T}\frac{\beta_{t}\text{I}}{\gamma^{2}\alpha_{t}\beta_{t-1}}\left(\mu_{\varphi }-\mu_{q}\right)\right]\;\;=\underset{\varphi }{\arg\min}\frac{\beta_{t}}{2\gamma^{2}\alpha_{t}\beta_{t-1}}\left[{\left\|\mu_{\varphi }-\mu_{q}\right\|}_{2}^{2}\right]$$

The mean parameter $\mu_{\varphi }\left(x_{t}^{\text{HR}},x^{\text{HR}},x^{\text{LR}},t\right)$ is parameterized as follows:

(11) $$\mu_{\varphi }\left(x_{t}^{\text{HR}},x^{\text{HR}},x^{\text{LR}},t\right)=\frac{\beta_{t-1}}{\beta_{t}}x_{t}^{\text{HR}}+\frac{\alpha_{t}}{\beta_{t}}g_{\varphi }\left(x_{t}^{\text{HR}},x^{\text{LR}},t\right)$$

where $g_{\varphi }(\cdot )$ is a NN approximating the diffusion trajectory of the forward process. After substituting it into (10), the final loss function is achieved as follows:

(12) $$\hat{\varphi }=\underset{\varphi }{\arg\min}{\left\|g_{\varphi }\left(x_{t}^{\text{HR}},x^{\text{LR}},t\right)-x^{\text{HR}}\right\|}_{2}^{2}$$

The constant parameters were dropped, as experiments demonstrated that this improves the model’s performance [23, 26]. In addition to the data fidelity $\ell_{2}$ loss, a learned perceptual image patch similarity (LPIPS) $\ell_{p}$ loss [29] was employed. The overall optimization function is given by:

(13) $${𝓛}_{\varphi }=\lambda {\left\|g_{\varphi }\left(x_{t}^{\text{HR}},x^{\text{LR}},t\right)-x^{\text{HR}}\right\|}_{2}^{2}+\ell_{p}\left(x_{t}^{\text{HR}},x^{\text{HR}}\right)$$

where λ is a hyper-parameter controlling the relative importance and we set it to 10 in this study.

### 2.3 Noise scheduler

**Figure 2:**
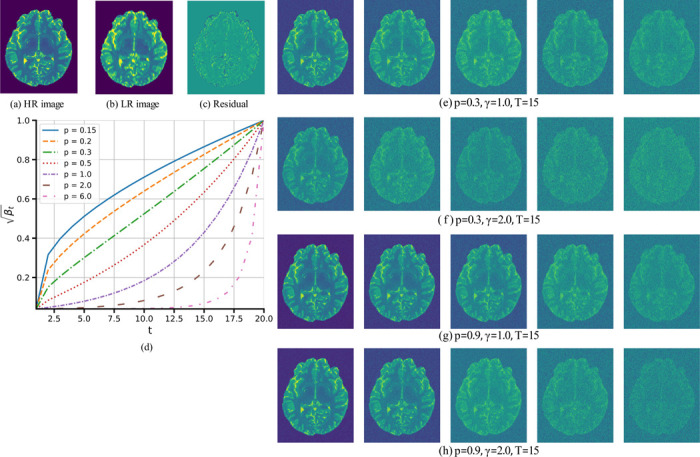
Residual shift denoising diffusion process. (a) shows the HR image, $x^{\text{HR}}$; (b) displays the corresponding LR image, $x^{\text{LR}}$; and (c) illustrates the residual error, $e_{0}=x^{\text{LR}}-x^{\text{HR}}$. (d) presents the evaluation of the noise scaling factor, $\sqrt{\beta_{t}}$, as a function of the diffusion time step, t. Panels (e)–(h) demonstrate the forward diffusion process driven by the residual error shift for different hyper-parameter sets.

This study utilizes a hyper-parameter γ and a noise scheduler ${\beta_{t}}_{t=1}^{T}$ in the forward diffusion process. Given that $\sqrt{\beta_{t}}$ and the scaling factor γ in (1) control the forward process, and it has been shown that a NN can approximate the forward diffusion trajectory [24, 26], $\gamma \sqrt{\alpha_{t}}$ needs to be small; thus, we set it to 0.04, which ensures that $q\left(x_{1}^{\text{HR}}|x^{\text{HR}},x^{\text{LR}}\right)\approx q\left(x^{\text{HR}}\right)$. Additionally, we set $\beta_{1}={\left(0.04/\gamma \right)}^{2}$ and used γ=2 to satisfy the first bounding condition $\beta_{1}\to 0$ (see Figure 1) and $\beta_{T}=0.9999$ to satisfy the second bounding condition $\beta_{T}\to 1$. We employed a non-uniform geometric noise scheduler proposed by Yue *et al.* [23] for $\sqrt{\beta_{t}}$ as follows:

(14) $$\sqrt{\beta_{t}}=\sqrt{\beta_{1}}\exp\left[{\left(\frac{t-1}{T-1}\right)}^{p}\log\sqrt{\frac{\beta_{T}}{\beta_{1}}}\right],t\in [2,T-1]$$

where the hyper-parameter p controls the growth rate, as shown in Figure 2. We used p=0.3 in our study, similar to a recent study [23]. Furthermore, we used 15 steps for training and four steps for sampling.

The Res-SRDiff model was implemented using PyTorch (version 2.5.1) and executed on NVIDIA A100 GPUs. The model was trained for 182,000 and 131,000 steps on the brain and prostate datasets, respectively, using a batch size of 16. The network was optimized with the Rectified Adam (RAdam) optimizer [30] and employed a cosine annealing learning rate scheduler [31]. The initial learning rate was set in the range of 2 × 10^−5^ to 5 × 10^−5^, and it was adjusted according to a cosine decay schedule throughout training. A warm-up phase of 5,000 steps was applied before transitioning to the cosine decay schedule to stabilize early training dynamics.

### 2.4 Patient data acquisition and data preprocessing

We used institutional ultra-high 7T brain T1 MP2RAGE maps [32] and publicly available axial T2w prostate cancer data [33] to train and evaluate the proposed method.

Our institutional dataset comprises 142 cases, which were divided into two non-overlapping sets: a training set (121 cases, 14,566 slices) and a test set (21 cases, 2,552 slices). This retrospective study was approved by the Mayo Clinic IRB. The institutional data were acquired using a 7 T Siemens MAGNETOM Terra with 8-channel transmit/32-channel receive head coil with the following key imaging parameters: TR = 4.5 s, TE = 2.2 ms, TI1/TI2 = 0.95/2.5 s, FA1/FA2 = 6°/4°, FOV = 230 × 230 cm^2^, matrix size of 288×288, a resolution of 0.8×0.8×0.8 mm^3^, and a total acquisition time of 8:44 min. FSL BET[34] was used to extract the brain mask from image inversion 1, which was subsequently applied to the T1 maps to remove the noisy background and skull. The T1 maps were down-sampled by a factor of 4^3^, resulting in a voxel size of 2.4 × 2.4 × 2.4 mm^3^ (a 4-fold reduction in each direction).

We randomly selected data from 334 patients in the public prostate dataset, which were split into two non-overlapping sets: a training set (268 patients, 10,480 slices) and an evaluation set (66 patients, 2,668 slices). The T2w MR images were acquired using a 1.5 T Siemens scanner with the following parameters: TR = 2.2 s, TE = 202 ms, FA = 110°, matrix size of 256 × 256, an in-plane resolution of 0.66 × 0.66 mm^2^, and a slice thickness of 1.5 mm. The T2w MR images were down-sampled by a factor of 18, yielding a voxel size of 2 × 2 × 3 mm^3^ (a 9-fold reduction in-plane and a 2-fold reduction along the slice axis).

Under-sampling of ultra-high B_0_ brain T1 maps and the axial T2w prostate images were performed in image space using the SimpleITK.Resample (version 2.1.1) package [35].

### 2.5 Quantitative and statistical analysis

We evaluated our method against four benchmark approaches: Bicubic, Pix2pix [36], CycleGAN [37], and TM-DDPM, which is a conventional DDPM with a vision transformer backbone [18]. All methods were trained for the same number of steps and with similar training parameters, except that the DDPM model had approximately three times as many training parameters.

The reconstructed HR image quality was quantitatively evaluated using four metrics: peak signal-to-noise ratio (PSNR), structural similarity index (SSIM)[38], gradient magnitude similarity deviation (GMSD)[39], and LPIPS [40]. Higher SSIM and PSNR values, and lower GMSD and LPIPS values, indicate better image restoration performance. PSNR quantifies the residual error between the restored and ground truth images, and its logarithmic scale aligns better with human perceptual judgments [41]. Furthermore, SSIM, GMSD, and LPIPS provide measures of the structural similarity between the restored images and the HR ground truth images.

Two statistical tests were employed to assess the significance of differences: a one-way analysis of variance (ANOVA) and Tukey’s honestly significant difference (HSD) test. Prior to these analyses, the Shapiro–Wilk test was conducted to evaluate the normality of the residuals. When the normality assumption was not satisfied, non-parametric methods were used, specifically the Kruskal–Wallis test followed by Dunn’s test with Bonferroni correction for multiple comparisons. The ANOVA tested the null hypothesis that the mean values for each method are equal, while the Kruskal–Wallis test assessed whether the distributions of the groups differed significantly. Tukey’s HSD and Dunn’s test with Bonferroni correction were then used to identify which specific pairs of groups differed significantly. For all analyses, the significance level was set at p<0.05.

## 3 Results

### 3.1 Brain T1 maps

**Figure 3:**
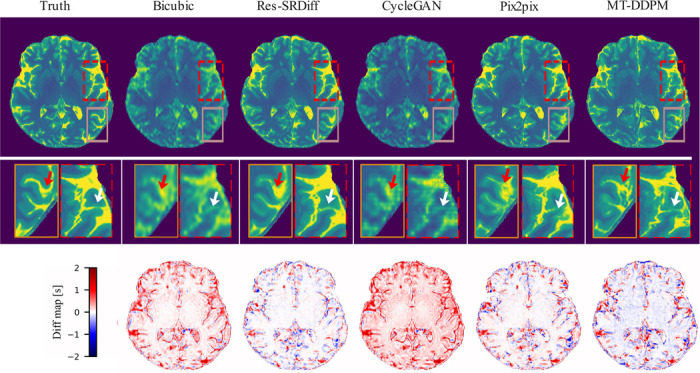
Qualitative results of the ultra-high field brain T1 MP2RAGE maps. The first row shows the ground truth image along with the restored outputs from our proposed Res-SRDiff and comparative models. The second row displays the zoomed-in regions corresponding to the dashed red and brown boxes. The white and red arrows highlight regions where our method outperforms the comparative models. The last row presents the difference map between the restored images and the ground truth.

The proposed method demonstrated superior performance, yielding lower residual errors and higher structural similarity, as illustrated in Figure 3 (first row). The zoomed-in panels (Figure 3 second row), highlighted by white and red arrows, show that our method more effectively captured fine details compared to the baseline methods. This observation aligns with the quantitative results, where our method achieved higher SSIM values and lower GMSD scores (refer to Table 1). Additionally, the reduced global residual error presented in the third row of Figure 3 suggests a closer agreement between the outputs of our method and the ground truth images.

In terms of computational efficiency, the average evaluation time for our proposed method was 0.46 ± 0.21 second per slice, which was markedly lower than that of the MT-DDPM method, with an evaluation time of 66.84 ± 27.72 seconds per slice. The quantitative metrics failed the Shapiro–Wilk normality test (with p-values ≪ 0.001); thus, we performed the non-parametric Kruskal–Wallis and Dunn’s tests. The Kruskal–Wallis test indicated statistically significant differences between the methods (p-values ≪ 0.0001) for all metrics. On average, our method outperformed all comparative methods in terms of all quantitative metrics, with statistically significant differences (p-values ≪ 0.001), except for LPIPS, where the difference with the Pix2pix method was not statistically significant (p = 0.08).

### 3.2 Pelvic T2w images

**Figure 4:**
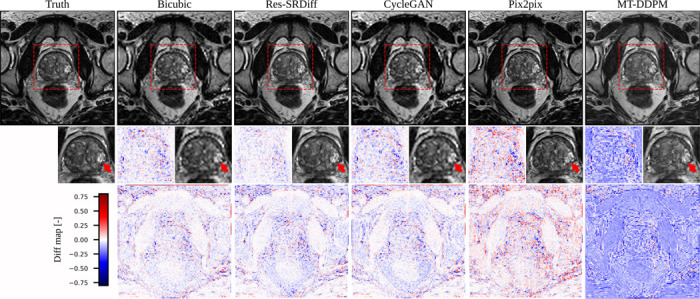
Qualitative results of the pelvic axial T2w images. The first row presents the ground truth image along with the restored outputs from our proposed Res-SRDiff and comparative models. The second row shows the zoomed-in regions outlined by the red dashed lines, where the red arrows indicate lesions that are visually restored closer to the ground truth by our method. The last row depicts the difference map between the restored images and the ground truth.

We compared our proposed Res-SRDiff model against Bicubic, CycleGAN, Pix2pix, and MT-DDPM. Our proposed method was able to restore axial T2w pelvic images with improved fidelity to the HR ground truth, as shown in Figure 4. Although the Pix2pix method successfully restored HR images that were globally similar to the ground truth, our method better restored the lesion, as indicated by the red arrow in the second row of Figure 4. These findings are further confirmed by the difference maps shown in the third row of Figure 4, where our method exhibits the smallest residual error compared with the other methods.

The evaluation time of our proposed method was 0.95±0.74 second per slice, which is substantially lower than that of MT-DDPM, with a validation time of 20.66±14.00 seconds per slice. The quantitative metrics failed the Shapiro–Wilk normality test (with p-values ≪ 0.001); thus, we performed non-parametric Kruskal–Wallis and Dunn’s tests. The Kruskal–Wallis test yielded p-values ≪ 0.0001, indicating that the differences between the methods were statistically significant for all quantitative metrics. Specifically, our method achieved the highest PSNR (27.72 ± 2.26) and the lowest GMSD (0.08 ± 0.02). Although our method, on average, achieved the second-best LPIPS after Pix2pix, the difference was not statistically significant (p=0.17). Table 1 summarizes the quantitative metrics and indicates whether the differences are statistically significant.

## 4 Discussion

MRI remains one of the most versatile modalities in both clinical practice and research due to its excellent soft-tissue contrast and ability to generate multiple image contrasts without ionizing radiation. However, the inherently long acquisition times can lead to patient discomfort and motion artifacts [42], often forcing a trade-off between spatial resolution and acquisition efficiency. One of the easiest approaches to mitigate these challenges is to increase the voxel size, but this can adversely affect the diagnostic quality [43] by introducing partial volume effects.

In this study, we introduced **Res-SRDiff**, an efficient probabilistic diffusion model designed to reconstruct high-resolution (HR) MRI images from low-resolution (LR) inputs. By leveraging the residual error, $e_{0}$, between the LR and HR images in the forward diffusion process, our approach shifts the HR image distribution toward that of the LR images. This enables the reverse process–implemented via a NN to accurately recover fine image details in only four sampling steps, markedly reducing the reconstruction time to under **one second per slice** compared with conventional diffusion models, which may require up to 20 seconds per slice.

Our experiments on both brain T1 maps and pelvic T2w images demonstrate that Res-SRDiff not only improves computational efficiency but also preserves critical anatomical details. For the brain T1 maps, qualitative assessments (as indicated by the white and red arrows in Figure 3) reveal that our method recovers fine structures with smaller residual errors compared to competing models. Quantitatively, our approach consistently achieved the highest PSNR and lowest GMSD, with statistically significant improvements (p≪0.05). Moreover, the small standard deviation observed across test samples suggests that incorporating the residual error $e_{0}$ contributes to a more stable and robust reconstruction process.

Similarly, in the pelvic T2w images, Res-SRDiff successfully reconstructs HR images with improved lesion depiction. Unlike the TM-DDPM method–which tended to exaggerate lesion sizes, possibly due to its progressive sampling process–our method maintained more anatomically accurate representations while also exhibiting lower residual errors. These findings align with the previous study that reported that DDPMs tend to generate blurry images [44]. The consistency of these results across both datasets underscores the advantage of integrating residual error information into the diffusion process.

Looking forward, several promising research avenues arise from our work. Expanding the Res-SRDiff framework to include other imaging modalities and incorporating it into real-time clinical workflows could remarkably enhance its effectiveness. Additionally, further refinements to the diffusion process, such as adaptive noise scheduling [45] or hybrid loss functions [46], may offer additional gains in image quality and reconstruction speed.

## 5 Conclusions

The proposed Res-SRDiff marks a substantial advancement in the creation of efficient diffusion-based super-resolution models for MRI. By minimizing the number of necessary sampling steps and utilizing residual error information, our approach achieves superior image restoration performance while ensuring both computational efficiency and consistency across a range of datasets.

Res-SRDiff provides a highly efficient and precise framework for MRI super-resolution, offering a notable reduction in computational time while maintaining or even exceeding the image quality of state-of-the-art methods. The integration of residual error shifting within the diffusion process signifies a meaningful step forward in medical image reconstruction, with potential implications for accelerating high-quality imaging in both clinical workflows and research applications.

## Figures and Tables

**Table 1:** Quantitative comparison of super-resolution models on two datasets: Axial T2w pelvic MRI and 7T brain T1 MP2RAGE maps. Results are presented as mean ± standard deviation for our proposed Res-SRDiff and comparative models. Bold values highlight the best-performing results, while underlined values indicate the second-best performance. Arrows indicate the direction of better results.

	Pelvic T2w MRI				7T brain T1 MP2RAGE map			
		
Models	PSNR [dB] ↑	SSIM [−] ↑	GMSD [−] ↓	LPIPS [−] ↓	PSNR [dB] ↑	SSIM [−] ↑	GMSD [−]↓	LPIPS [−] ↓

Bicubic	25.47_±2.61_	**0.75**_**±0.06**_ ∗	0.10_±0.02_	0.69_±0.15_	22.00_±1.37_	0.31_±0.16_	0.12_±0.02_	0.38_±0.07_
cycleGAN	25.84_±1.96_	0.73_±0.05_	0.10_±0.01_	0.45_±0.10_	21.89_±1.09_	0.86_±0.02_	0.12_±0.02_	0.21_±0.05_
Pix2pix	24.83_±2.09_	0.66_±0.05_	0.11_±0.01_	**0.20**_**±0.05**_ ∗	24.63_±1.32_	0.90_±0.03_	0.10_±0.02_	0.09_±0.04_ ∗
TM-DDPM	25.12_±4.46_	0.73_±0.16_	0.13_±0.04_	0.51_±0.49_	23.22_±5.02_	0.85_±0.13_	0.12_±0.05_	0.25_±0.10_
Res-SRDiff	**27.72**_**±2.26**_	**0.75**_**±0.05**_	**0.08**_**±0.02**_	0.21_±0.11_	**26.28**_**±1.41**_	**0.92**_**±0.03**_	**0.07**_**±0.02**_	**0.08**_**±0.02**_

∗ denotes results that are not statistically significant based on the multi-comparison test (p-value > 0.05).

## Data Availability

The ProstateX data is publicly available at the TCIA portal (https://www.cancerimagingarchive.net/analysis-result/prostatex-seg-hires/). Our institutional data cannot be made publicly available upon publication because they contain sensitive personal information.
